# A multiwell plate-based system for toxicity screening under multiple static or cycling oxygen environments

**DOI:** 10.1038/s41598-021-83579-1

**Published:** 2021-02-17

**Authors:** Ming Yao, Glenn Walker, Michael P. Gamcsik

**Affiliations:** 1 Department of Mechanical and Aerospace Engineering, NC State University, Raleigh, USA; 2 UNC/NCSU Joint Department of Biomedical Engineering, Campus Box 7115, Engineering Bldg 3, 1840 Entrepreneur Drive, Raleigh, NC 27695-7115 USA; 3 Department of Biomedical Engineering, University of Mississippi, Oxford, MS USA

**Keywords:** High-throughput screening, Cancer microenvironment, Cancer models, Assay systems, Chemotherapy, Drug development

## Abstract

Tumor tissue contains a continuous distribution of static and dynamically changing oxygen environments with levels ranging from physiologically normal oxygen down to anoxia. However, in vitro studies are often performed under oxygen levels that are far higher than those found in vivo. A number of devices are available to alter the oxygen environment in cell culture, including designs from our laboratory. However, in our devices and most other designs, changing the media in order to feed or dose cells remains a disruptive factor in maintaining a consistent hypoxic environment. This report presents a novel 96-well plate design that recirculates the local oxygen environment to shield cells during media changes and facilitates toxicity studies of cells cultured under varying oxygen levels. The principle behind the design is presented and the response of human pancreatic cancer PANC-1 cells treated with tirapazamine and doxorubicin under eight different static or cycling oxygen levels was measured. As expected, tirapazamine is progressively more toxic as oxygen levels decrease but retains some toxicity as oxygen is cycled between hypoxic and normoxic levels. Doxorubicin sensitivity is largely unaffected by changing oxygen levels. This technology is ideal for assessing the effects of oxygen as a variable in toxicity screens.

## Introduction

Studies show that at least 50–60% of locally advanced human tumors contain regions of hypoxic or anoxic tissues^1,2^. The median oxygen levels in human tumors originating from 14 different tissues expressed as oxygen partial pressure (*p*O_2_) range from levels of 2 mmHg in pancreatic adenocarcinoma to 32 mmHg in rectal carcinomas^1^ which is far below the levels found in healthy tissue (~ 50–70 mmHg)^3^. Hypoxic regions vary both spatially and temporally, resulting in an infinite landscape of oxygen levels and cycles that can be present in any given tumor. Hypoxia can inhibit cellular differentiation and help maintain cancer stem cells^4^, drives cancer progression^5–7^, results in therapy resistance^8,9^ and is a widely observed negative prognostic indicator^10^. Compared to chronic hypoxia, cycling hypoxia is thought to be a more effective driving force in cancer progression, resulting in more aggressive tumor cells^11,12^. In vitro studies show that the response of cancer cells to chemotherapeutic drugs is changed under static hypoxic conditions^13,14^ but few studies have probed response under cycling hypoxia.

Static or cycling hypoxia studies in cultured cells are often conducted using standard cell culture ware in sealed chambers and require the use of a glove box to feed or add drug to cells in order to prevent undesired changes in pericellular oxygen levels^15,16^. These chambers and glove boxes require large gas volumes to flush and equilibrate the media, often probe only a single oxygen level, and are unable to perform studies requiring rapid (< 30 min) cycles of hypoxia. Cycling hypoxia in tumors is caused by variations in red blood cell flux, and occurs with periodicities on the order of minutes to hours to days^11^ and standard approaches can sample only a fraction of these conditions. Microfluidic devices offer shorter diffusion distances, multiplexing capability and lower gas volumes to address all of these limitations^17,18^, but are not easily adopted by oncology laboratories. Alternatively, standard cell culture ware with well inserts^19,20^ or gas-permeable bottoms^21–23^, offer better control of static oxygen levels and rapid cycling and can be more readily incorporated into existing protocols using standard laboratory equipment. However, changing media or dosing cells with these designs has not been demonstrated. Because of this limitation, few studies have systematically probed multiple oxygen environments and cycling conditions on drug response in tumor cells, which has inhibited the development of agents that can be targeted to the most aggressive cancers.

We previously demonstrated two different 96 well plate designs that can culture cells under static and dynamic hypoxia^24,25^. However, in both designs, feeding or adding drugs to the wells exposed the cells to normoxia and it would typically take ~ 15 min to re-equilibrate the cells to hypoxia. This change in oxygen levels may stress the cells and influence drug response. In this report, a modification of our both our previous technologies^24,25^ was designed, fabricated and tested to enable cell feeding and/or drug dosing with minimal change in the pericellular *p*O_2_ environment in all eight rows of a 96-well plate without the use of a glove box. This design uses a novel gas recirculation route to shield the cells during the media change and greatly facilitates the study of drug response of cultured cells under varying static and cycling *p*O_2_ levels.

Using our new design, we measured the drug dose–response of PANC-1 pancreatic carcinoma cells to tirapazamine and doxorubicin cultured under eight different static and eight different cycling *p*O_2_ levels. Compared to cells under *p*O_2_ = 139 mmHg (‘normal’ incubator conditions, i.e. normoxia), PANC-1 cells show progressively greater sensitivity to tirapazamine as static oxygen levels decrease. Interestingly, PANC-1 cells retained sensitivity to tirapazamine under cycling oxygen conditions where cells are exposed to non-hypoxic environments for half of the cycle. In contrast, PANC-1 sensitivity to doxorubicin showed little change under a range of static or cycling oxygen environments. This new device offers a screening approach to determine the toxicity response under varying oxygen conditions.

## Results

### Design principle

**Figure 1:**
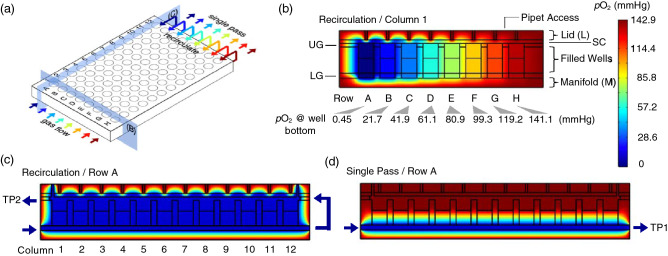
Plate Design and Modeling. (**a**) Diagram of the plate cross-sections used to mathematically model the oxygen distribution. Arrows show the direction of gas flow and are color-coded to represent the *p*O_2_ levels. (**b**) Heat map of the predicted oxygen distribution generated across eight rows A–H in Column 1 of media-filled wells by delivery of eight different gas mixtures recirculated from the lower to the upper channels. Labels indicate the acrylic lid (L) and manifold (M), the silicone cover (SC) and the upper and lower channels formed by the rubber upper gasket (UG) and lower gasket (LG), respectively. The predicted pO_2_ levels at the bottom of wells in each row are listed below the row letter. (**c**) Heat map of predicted oxygen distributions along Row A when gas is recirculated and (**d**) in Row A when gas is in single-pass mode. Oxygen sensors measure the *p*O_2_ levels at test points 1 (TP1) and 2 (TP2).

The objective of this work is to provide a way to remove and add media or drug to wells in a 96-well plate while maintaining eight different hypoxic conditions without the use of a glove box. The principle behind this device is shown in Fig. 1a. Using the same mixing tree design as in our earlier publication^25^, and two feed gases (95% air/5%CO_2_ and 95% N_2_/5%CO_2_), eight different static or cycling gas mixtures are delivered to channels below each row (A-H) of a permeable bottom 96-well plate. A 125 μm thick gas-permeable polydimethylsiloxane (PDMS) membrane that forms the bottom of each well. Gas mixtures delivered from below the membranes only has to diffuse 125 μm to the cells grown on the upper surface so the pericellular environment is dominated by the oxygen content of this gas. In the configuration shown in Fig. 1a, a low oxygen mixture (*p*O_2_ = 0 mmHg) is delivered beneath the wells in Row A with the *p*O_2_ level delivered to each row increasing by ~ 20 mmHg up to Rows H (*p*O_2_ = 141 mmHg). Unlike our earlier designs, this new device recirculates the gas from beneath the plate to channels above the plate to effectively blanket the top surface of the wells with the same *p*O_2_ level delivered from below. This design shields the cells from the ambient atmosphere, whether biological safety cabinet or incubator, and maintains cells under any desired oxygen environment while media is removed and added. In addition, when pipet access to the wells is not needed, the device can be operated in single pass mode where the gas passing beneath the wells is vented to the surroundings (Fig. 1a).

### Computational modeling

Mathematical modeling is key to designing and predicting the performance of a recirculating plate. In order to recirculate gases and maintain pipet access to the culture wells, an acrylic lid with access holes and a PDMS cover are used in this new design. Computational modeling is necessary to determine whether the mass transport properties of these new components provide an adequate gas barrier to shield the cells during media changes or drug dosing. This new device uses the same mixing tree from our earlier design^25^ that combines two feed gases that, when warmed and humidified, can deliver eight different *p*O_2_ levels flowing through the lower channels beneath the wells in Rows A to H^25^. Gas flow arrows in Fig. 1a are color-coded to represent the eight different *p*O_2_ levels within that range.

Modeling was used to predict oxygen mass transfer capabilities across the eight different cell culture media-filled wells in Column 1 and to the twelve wells in Row A of the plate with the new lid and cover in place. The cross-sections of the columns and rows that were modeled are shown by the blue planes in Fig. 1a. Row A was chosen for modeling as this is the most hypoxic environment on the plate and best demonstrates the capability of the device to shield the cells from the ambient laboratory atmosphere. In recirculating mode, the predicted oxygen levels across the device are shown in the heat map in Fig. 1b for the eight rows in Column 1 shown in cross-section. This heat map indicates that the media in the wells has a uniform oxygen content from top to bottom and that the flowing gas and 1 mm pipet access holes and underlying PDMS cover (PC) provide sufficient isolation of the wells and media from the surrounding ambient atmosphere. This model predicts that cells grown in Rows A to H will be exposed to pericellular *p*O_2_ levels between 0.5 and 141 mmHg at the well bottoms. As gas flows at a rate of 25 mL/min down the channel beneath Row A wells, the oxygen content of the media in the wells in Columns 1–12 remains unchanged ensuring that the oxygen content at this gas flow rate is uniform down the length of the row as gas is recirculated (Fig. 1c). The *p*O_2_ levels exiting the channels at the top of the plate were measured at test point 2 (TP2, Fig. 1c) to determine if the gas content is lost during the recirculation circuit (see below). In single pass mode, the bottom of the wells is exposed to the gas delivered from below while the upper surface equilibrates with the surrounding ambient atmosphere generating a vertical oxygen gradient in each well (Fig. 1d). This gradient is stable as long as the oxygen conditions at the bottom of the well remain dominated by the gas mixture delivered from below^25^. Table 1 shows the predicted *p*O_2_ levels in wells in both recirculating and single pass modes. The model was also used to predict the oxygen content of the gas exiting the device in single pass mode that were measured at test-point 1 (TP1, Fig. 1d) (see below) and these predictions are also listed in Table 1.

**Table 1:** Predicted, measured and calculated *p*O_2_ Levels at 37 °C.

	A	B	C	D	E	F	G	H
**Recirculate mode**								
Predicted @ 100 μm	0.45	21.6	41.8	61.1	80.0	99.3	119.2	141.0
Measured @ 100 μm	0.35 ± 0.17	20.0 ± 0.4	40.3 ± 0.2	60.1 ± 0.1	79.0 ± 0.6	96.0 ± 0.3	114.0 ± 0.4	137.3 ± 0.6
Measured @ TP2	0	20.4 ± 0.4	40.8 ± 0.4	60.4 ± 0.5	79.0 ± 0.5	95.5 ± 0.5	115.0 ± 0.6	137.4 ± 2.0
Calculated Pericellular *p*O_2_	0.2	19.9	41.0	60.4	79.4	96.6	114.3	138.0
**Single Pass Mode**								
Predicted @ 100 μm	3.9	24.9	44.6	63.2	81.6	100.4	119.9	141.0
Measured @ 100 μm	3.7 ± 0.4	21.8 ± 0.7	41.4 ± 0.8	65.1 ± 0.9	83.9 ± 0.5	104.7 ± 1.4	121 ± 1.4	139.2 ± 1.4
Predicted @ TP1	0.42	21.6	41.8	61.1	80.0	99.4	119.2	141.0
Measured @ TP1	0	19.7 ± 0.4	41.6 ± 1.8	60.6 ± 0.7	79.8 ± 1.3	97.2 ± 1.8	114.6 ± 0.4	138.8 ± 0.6
Calculated Pericellular *p*O_2_	1.9	20.5	41.5	62.9	81.9	101.0	118.0	139.0

### Device fabrication

**Figure 2:**
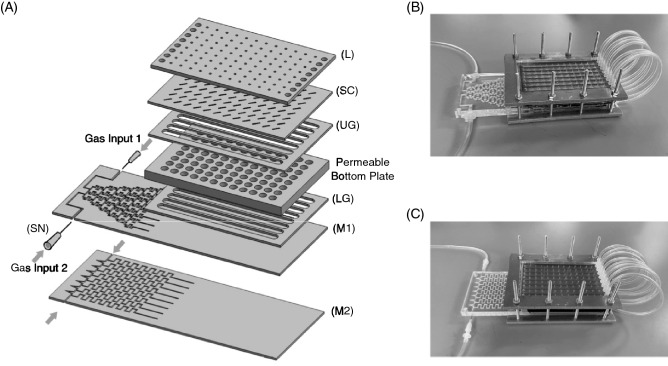
(**A**) A diagram showing the components of the device. The letters correspond to the structures shown in Fig. 1 with the addition of two syringe needles (SN) to connect the gas feeds. Two different manifolds are used. The mixing tree manifold (M1) is used to screen response to eight different oxygen environments. The gas partitioning manifold (M2) supplies one oxygen environment to Rows A-D and a second oxygen environment to Rows E–H. (**B**) Shows the assembled device with manifold M1 and (**C**) the assembled device with manifold M2. The tubing loops recirculates the gas flow from below the permeable bottom wells in each row to over the top of the media.

Based on the dimensions and materials evaluated in the models, the device was fabricated and shown in an exploded view in Fig. 2A. Acrylic was used for the manifold (M1, M2) and lid (L) and Buna-N rubber was used in fabricating the lower (LG) and upper (UG) gaskets that form the lower channels beneath and upper channels above the wells, respectively. The mixing tree manifold (M1) provides eight different static or cycling *p*O_2_ levels to each row of the plate as demonstrated previously^25^. The second gas-partitioning manifold design (M2) that was characterized in an earlier study^24^ and is used here to deliver one static or cycling *p*O_2_ level to Rows A-D and another static or cycling *p*O_2_ level to Rows E–H by providing precisely balanced flow of the two input gases. The devices are held together by a metal clamp and the gas flow is redirected from below to above the plates by eight flexible tubing loops that can be connected and disconnected for recirculating or single pass of gas flow modes, respectively (Fig. 2B,C). The assembled device with manifold M1 is shown in Fig. 2B and manifold M2 is shown in Fig. 2C. The device is switched to the recirculating mode only during media changes or drug dosing in order to reduce water evaporation due to gas flowing above the wells^25^.

### Experimentally measured oxygen levels match predicted levels

The performance of the device was first evaluated by measuring oxygen levels at the output of the manifold in single pass mode at test point 1 (TP1, Fig. 1d) and comparing the data to the model predictions (Table 1). The readings match well across each row and demonstrate that the model is valid, and the gaskets provide good isolation in each lower channel from the surrounding environment and from adjacent channels. Also shown in Table 1 are the measurements taken after recirculating the gas to the upper channel and obtaining readings at the outlet (TP2, Fig. 1c). These data also show that the system is gas-tight as there is little change in gas mixture content as the gas recycles from below (TP1) to above (TP2).

The measured *p*O_2_ values in 200 μL of media in the wells at 37 °C using humidified gases in recirculating and single pass modes are shown in Table 1. Due to the fragility of the fiber optic probes, these measurements in the wells are obtained from a location 100 μm above the well bottom (see “Methods” section). The actual *p*O_2_ level at the well bottom where cells are cultured was calculated by averaging the values measured at 100 μm with the TP1 measurements. Since the gas that is measured at TP1 is separated from the cells by the 125 μm thick membrane, the top of the membrane, that is the pericellular location, is approximately half-way between these two measured points. The pericellular *p*O_2_ levels in Rows A to H were calculated and shown in Table 1 for both the recirculating and single pass modes. The device remains in single pass mode for most of the studies and these levels are what are reported in the experiments performed in this study. These levels vary by < 2 mmHg from our data presented earlier with the differences due to the use of 200 μL in this study compared to 300 μL used earlier^25^. Recirculating mode is only used for feeding or dosing cells. In this mode, the calculated *p*O_2_ levels shown in Table 1 are similar to single pass data with the biggest variation at the most hypoxic levels for cells in Row A.

**Figure 3:**
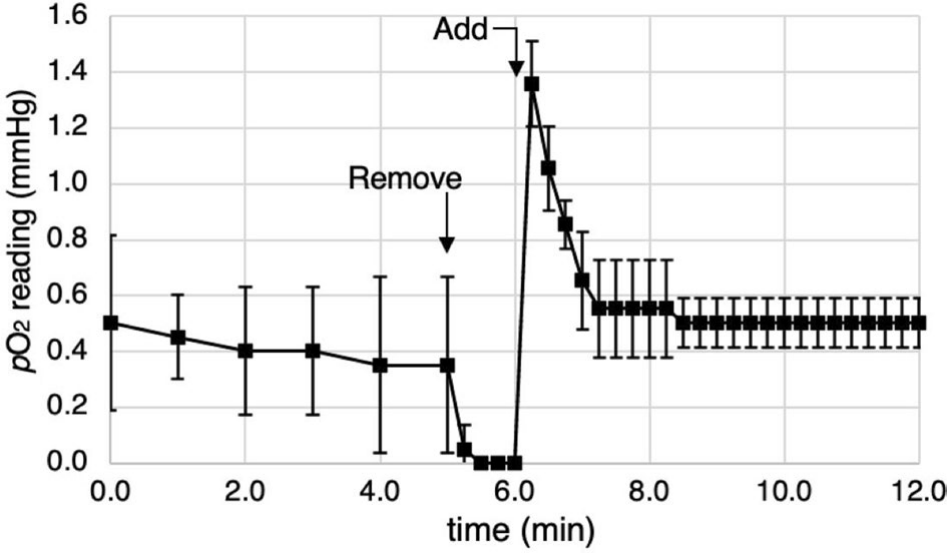
Time dependence of the oxygen levels measured at a point 100 μm above the membrane in the gas recirculation system when mimicking media change process with each well containing 200 μL of water. Water is removed at the 5 min timepoint and fresh pre-equilibrated water added at 6 min. The *p*O_2_ was measured in a well in Row A.

The ability of the assembled device to tolerate media changes or drug doses without compromising hypoxia was evaluated by assembling the device, recirculating humidified gases and placing the oxygen sensor in 200 μL of distilled water in a well in Row A as this row, being the lowest *p*O_2_ level, would be expected to be most affected by a media change. The *p*O_2_ level between t = 0 and 5 min was ~ 0.5 mmHg (Fig. 3). The water was then removed using a standard multichannel pipettor and the gas from the upper channel flooded the well, bringing the measured *p*O_2_ level to zero. Water pre-equilibrated to *p*O_2_ = 0.5 mmHg was then added and the sensor reading briefly spikes to ~ 1.4 mmHg but rapidly returns to a stable low reading within a minute. Overall, the *p*O_2_ levels are perturbed for ~ 2 min during the water change.

### PANC-1 cell growth is unchanged under a range of pO_2_ levels between 2 and 139 mmHg

Cells were seeded into the pre-warmed permeable-bottom 96-well plates in a standard biological safety cabinet (see “Methods” section). The cell-filled plate was placed in a standard air/CO_2_ incubator to allow cells to attach for 24 h. Plates were clamped onto the manifold and gas flow was then initiated from two input gas cylinders and the cells were grown under eight different static *p*O_2_ levels. As indicated in Table 1, the pericellular static *p*O_2_ levels range from 2 to 139 mmHg in the single pass mode. After 72 h, the cells were stained and counted or assayed using MTT. For counting, cell nuclei were stained and counted by image analysis as indicated in the Methods section. This counting method was validated by seeding wells with 5,000, 10,000 and 20,000 cells, and showing the resulting image-based cell counts yield the expected ratio of 1:2:4 under eight different static *p*O_2_ levels (Supplementary Material, Fig. S1). Cell counting determined that there was no difference in the growth rate of PANC-1 cells across the eight different static *p*O_2_ levels (Supplementary Material, Fig. S2). However, MTT staining increased steadily from 139 to 2 mmHg (Supplementary Material; Fig. S2). Therefore, MTT absorbance levels cannot be used to compare cell proliferation between cells grown under different oxygen conditions. MTT absorbance does track with initial cell seeding number under a given *p*O_2_ level under both static and cycling *p*O_2_ environments (Supplementary Material, Figs. S3, S4). Therefore, the drug dosing studies presented herein are normalized to the zero dose reading at a given *p*O_2_ level to correct for this oxygen-dependent MTT response.

Successful culture of cells in antibiotic-free media over 4 days, as demonstrated in Supplementary Material Fig. S2, demonstrated that the pipet access holes, sealed below by the PDMS cover (PC, Fig. 2A) provided sufficient protection from microbial contamination.

### Drug response varies across multiple oxygen environments

After cell seeding and attachment for 24 h under standard incubator air/CO_2_ conditions as described above, cells were equilibrated under the eight different static or cycling *p*O_2_ levels for 24 h. Just prior to drug dosing, the plate was switched to recirculating mode for 1 h. Since media pre-equilibrated at eight different *p*O_2_ levels is needed to feed or dose the cells, an identical ‘donor’ plate was filled with fresh media with or without drug and connected to the same gas mixtures and in recirculating mode to pre-equilibrate the media with the necessary eight different *p*O_2_ conditions before introduction to the cells. Two identical devices therefore are needed; one containing the cells cultured under the desired eight different oxygen conditions and another donor plate to pre-equilibrate the media under these eight different *p*O_2_ conditions.

**Figure 4:**
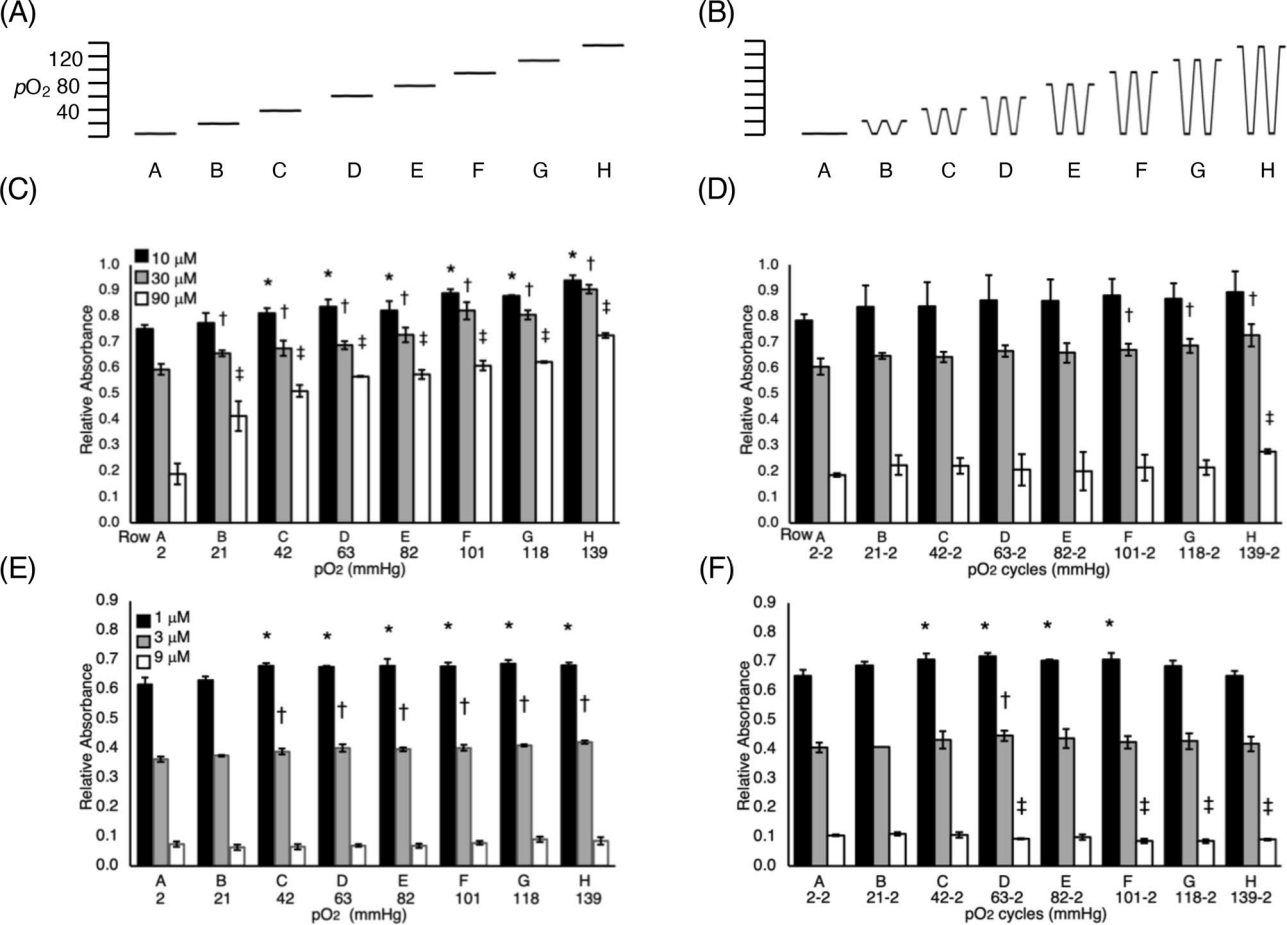
Drug dose response studies of PANC1 cells under (**A**) static *p*O_2_ levels delivered to the lower channels for each row and (**B**) and cycling *p*O_2_ levels with switching between *p*O_2_(hi) and *p*O_2_(lo) amplitudes indicated. (**C**) Dose response to tirapazamine under static and (**D**) cycling conditions. (**E**) Dose response to doxorubicin under static and (**F**) cycling conditions. *p < 0.05 relative to Row H at lowest dose; ^†^p < 0.05 relative to Row H at middle dose; ^‡^p < 0.05 relative to Row H at highest dose.

Using mixing manifold, M1 (Fig. 2B) eight different oxygen environments were delivered to each row of the 96-well plate^25^. The measured eight static *p*O_2_ levels delivered by manifold M1 to the channels beneath the cells are shown diagrammatically in Fig. 4A. In cycling experiments, all rows were exposed to a low *p*O_2_ (*p*O_2low_) level of 2 mmHg for 20 min followed by immediate switching to eight different high *p*O_2_ (*p*O_2hi_) levels ranging from 2 to 139 mmHg for another 20 min. These cycles were repeated for 24 h. The relative amplitudes of the different cycling patterns of gas delivered by the manifold M1 to the lower gas channels beneath each row is shown in Fig. 4B. Note that in Row A in Fig. 4B, the gas ‘cycles’ between *p*O_2low_ = 2 mmHg to *p*O_2hi_ level of 2 mmHg, so the *p*O_2_ level is essentially static. This is a convenient control and allows comparisons between sensitivity under static and cycling environments as gas delivery to Row A is equivalent in Fig. 4A,B.

On a 96-well plate with each of eight rows exposed to a different *p*O_2_ level, 12 wells are under identical oxygen environments. Therefore, in order to perform dose–response measurements in triplicate, four doses of drugs can be tested at each oxygen environment. For tirapazamine, the four doses tested were 0, 10, 30 and 90 μM and were selected based on the reported sensitivities of PANC-1 to tirapazamine (IC_50_ = 77 μM (normoxia), IC_50_ = 14 μM (hypoxia)^26^. Under static conditions, the pericellular *p*O_2_ values range from 2 mmHg (Row A) up to 139 mmHg (Row H) in this device. Due to the dependence of MTT metabolism on oxygen content (Supplementary Material, Fig. S2), the MTT readings at each *p*O_2_ level were normalized to the reading at zero dose at each *p*O_2_ level. The results in Fig. 4C show that PANC-1 cells were more sensitive to tirapazamine under low *p*O_2_ levels as the relative level of MTT staining progressively decreases with decreasing *p*O_2_ levels. At most doses, there are statistically different responses for the different oxygen levels compared to the data in Row A (*p*O_2_ = 2 mmHg).

The data for PANC-1 cells exposed to oxygen cycles and increasing doses of tirapazamine are shown in Fig. 4D. The amplitude of the cycles (*p*O_2low_-*p*O_2hi_) is given below the graph in Fig. 4D. For this drug, only the largest cycling amplitudes (Rows F to H) show any significant difference in response to the different drug doses when compared to Row A.

For doxorubicin, we examined the drug dose response under 0, 1, 3 and 9 μM, based on the reported doxorubicin IC_50_ (0.5–2 μM) for PANC-1 cells under normoxia^27,28^. Figure 4E shows the relative MTT staining of PANC-1 to doxorubicin across the different oxygen environments. Compared to Row A, drug sensitivity decreases slightly in Rows B-F, but the magnitudes of these differences are much less than those observed for tirapazamine. Under cycling conditions, only Row D shows significant difference at all doses compared to Row A. The largest cycling amplitude of 2–139 mmHg (Row H) is statistically identical at all doses when compared to Row A.

**Figure 5:**
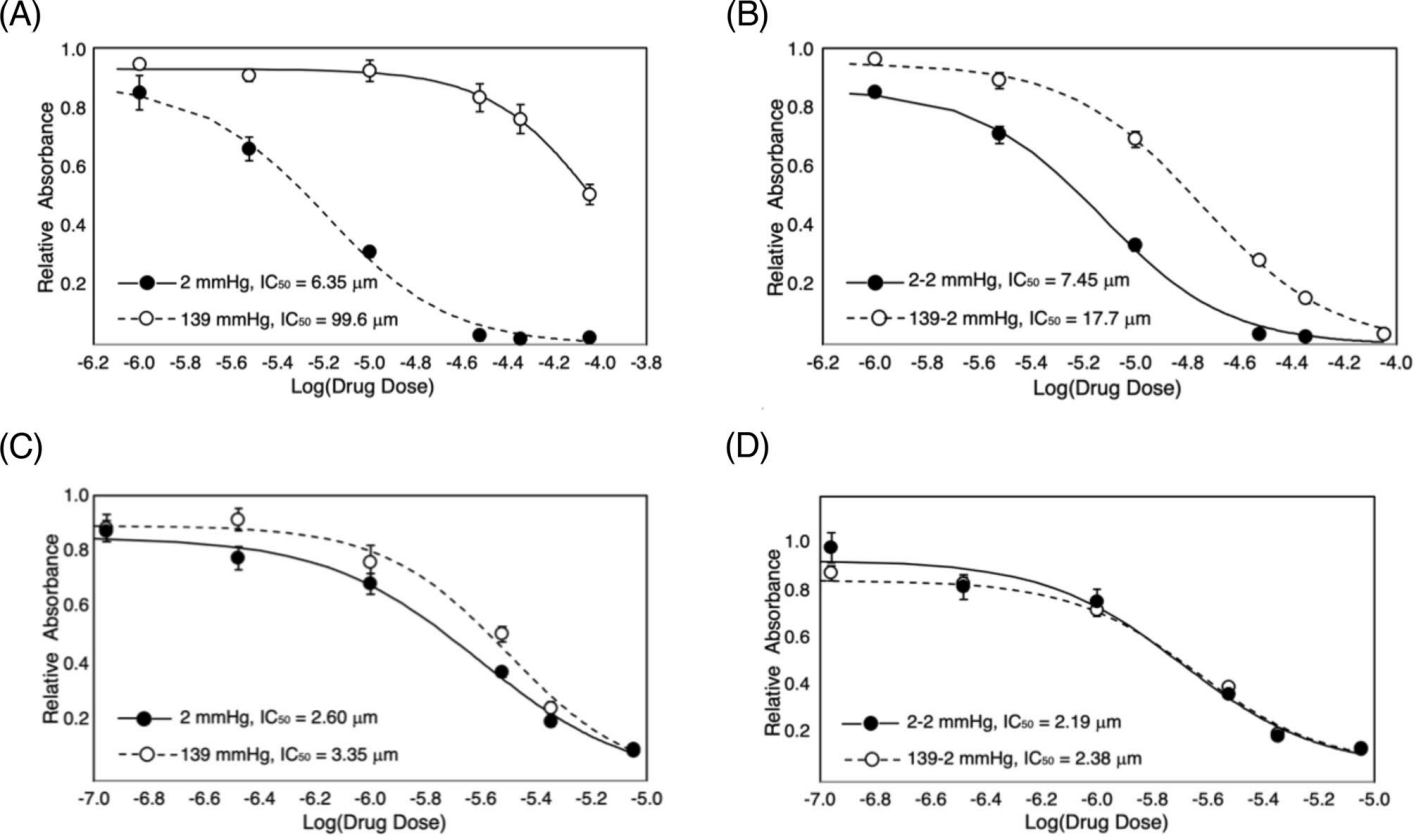
Drug dose response of PANC1 cells to (**A**) tirapazamine under static and (**B**) under cycling hypoxia and (**C**) doxorubicin under static and (**D**) cycling hypoxia conditions.

Although the data in Fig. 4 give an indication of relative sensitivity, the few doses tested are not sufficient to evaluate an IC_50_ with any confidence. Therefore, two of the *p*O_2_ levels tested were selected for a more detailed IC_50_ determinations using the second manifold design, M2 (Fig. 2C), that allows up to 16 doses in triplicate under one of two different *p*O_2_ levels. For tirapazamine, a total of seven doses of drug (including the zero dose control) were used to compare the IC_50_ at static levels of *p*O_2_ = 2 and 139 mmHg. Figure 5A shows that the IC_50_ decreases from 99.6 μM at 139 mmHg to 6.35 μM at 2 mmHg; an approximately 16-fold decrease. For cycling, the IC_50_ was determined at cycles of 2–139 mmHg and 2–2 mmHg (i.e. static) conditions. Figure 5B shows the 2–2 mmHg IC_50_ = 7.45 μM which increases as PANC-1 cells are exposed to cycles of 2–139 mmHg with an IC_50_ = 17.72 μM.

For doxorubicin, seven doses were tested at static *p*O_2_ levels of 139 mmHg and 2 mmHg. The data show statistically significant differences at many dose levels, but the fitting of the data shows only a small difference in IC_50_ values of 3.35 and 2.60 μM at 139 and 2 mmHg, respectively (Fig. 5C). Under the cycling *p*O_2_ level of 2–2 mmHg, the IC_50_ = 2.19 μM is similar to the value found for the 2–139 mmHg cycles with an IC_50_ = 2.38 μM (Fig. 5D). Both the tirapazamine and doxorubicin data shown in Fig. 5 are consistent with the four dose data shown in Fig. 4.

## Discussion

Drug screening is most often done under incubator conditions of *p*O_2_ ~ 140 mmHg which is much higher than the range found in healthy tissue (30–75 mmHg) or in tumors (30 – 0 mmHg)^3^. The widespread use of relatively gas impermeable polystyrene plates with cells cultured on the bottom surface below a few millimeters of growth media means that even in a standard incubator, the pericellular *p*O_2_ levels are likely lower than the applied atmosphere and constantly changing as cells grow and alter their oxygen consumption^29–31^. Even in hypoxia studies, usually only one low *p*O_2_ level is probed and cells are again cultured under a layer of media in impermeable plates, so the actual oxygen content is often unknown. Therefore, a comparison between ‘normoxia’ and ‘hypoxia’ drug response is difficult to interpret without a knowledge of the oxygen conditions at the cellular level. Permeable bottom plates are commercially available and designed to address this problem^21,23^. However, since tumors can display essentially an infinite range of static and cycling oxygen conditions, the drug response needs to be assessed under a range of conditions. Clinical studies have shown that a significant fraction of hypoxic tissue in patient tumors is a negative prognostic factor^10^ and more recent work suggest that cycling hypoxia is “…a more serious problem for cancer patient treatment…” than static hypoxia^12^. Unfortunately, therapy response studies under cycling hypoxia are rare due to the fact that the equipment (glove box, hypoxia chambers) are not readily amenable to rapid (< 30 min) gas exchange cycles that are one component of the changing oxygen landscape found in tumors^11^.

Our technologies were developed to be readily adaptable to current protocols used in oncology and pharmacology laboratories. The 96-well plate format is a familiar culture vessel that is widely used and amenable to standard multichannel pipettors and plate readers. The experiments outlined herein can be duplicated in microdevices^32^ but these devices are not routinely used in many laboratories. In earlier devices from our previous work, the pericellular *p*O_2_ levels in permeable bottom dishes/plates are dominated by the gas delivery from below the membrane^21,23,25^. This ‘open well’ design is simple to fabricate and easy to use and works well for profiling molecular content^25^ or kinetics of response to *p*O_2_ changes^24^. However, in those designs, if media is removed, the cells would be exposed to the incubator or biosafety cabinet *p*O_2_ and addition of fresh media, with or without drug, would take ~ 10–15 min^25^ to re-equilibrate at the desired oxygen level. The principle behind this new device was to protect cells from the surrounding environments by temporarily blanketing the media upper surface and cells with the same gas mixture that is delivered from below. Therefore, when media is removed, the cells do not experience a large shift in oxygen level. Our modeling and experimental measurements show that the small pipet access holes and PDMS cover shields the cells from the surrounding atmosphere. PDMS is highly permeable to oxygen but the combination of gas flow and small pipet access holes in the relatively impermeable acrylic lid provide a sufficient barrier to isolate each row of wells from the surrounding atmospheres and the gaskets are sufficient to prevent cross-talk between gas mixtures from neighboring rows. The pipet access holes were sized to allow the use of commercially available narrow pipet tips.

PANC-1 cell growth is unchanged under the range of static oxygen levels when determined by cell counting. However, as oxygen levels decrease, the MTT absorbance readings significantly increase for statistically identical cell numbers. This is likely due to the influence of oxygen on the processes that affect MTT metabolism. Therefore, using MTT as a comparative measure of PANC-1 cell number or proliferation between two different oxygen atmospheres would be misleading and these data serve as a caveat for the use of MTT for comparing cell growth or response in different environments. However, under a specific *p*O_2_ level, MTT staining tracks with PANC-1 cell number so this assay is valid as long as the oxygen level within a test group is unchanged. Studies of MTT and other proliferation assays in different cell lines as a function of oxygen environment are now underway.

This new technology is suitable for toxicity studies under static or cycling oxygen environments. As expected, tirapazamine is significantly more effective under low oxygen levels, and the sensitivity of PANC-1 cells to this drug progressively increases as the oxygen levels decrease. The 16-fold increase in sensitivity to tirapazamine on changing from normoxic to hypoxic conditions are comparable to the 6-fold^26^ or 14-fold^33^ changes observed previously.

Our earlier work showed that cycling oxygen, between *p*O_2_ levels of 135 and 1 mmHg, induced a 2.5-fold increase in HIF-1α levels, a 2.1-fold increase in glucose uptake and 4.0-fold increase in aldehyde dehydrogenase activities in the PANC-1 cell line compared to static hypoxia^24,25^. In this study, cycles of 139–2 mmHg produced a 2.4-fold increase but only a 1.1-fold difference decrease in the sensitivity of PANC-1 cells to tirapazamine and doxorubicin, respectively, compared to static hypoxia. Although the 139–2 mmHg amplitude cycle is not physiologically relevant, a sample of earlier studies show similar amplitudes are often used in cycling experiments^34,35^, and may provide a good test of the most extreme stress that can be applied to cells. However, cycles that are physiologically possible, such as those in Row B to E in Fig. 4D,F, show only small differences in drug sensitivity compared to static hypoxia in Row A. This may change as cycle periods or durations are changed. These parameters can easily be adjusted by altering the setting in the gas flow controllers. We will continue to focus our studies on static and dynamically changing oxygen environments that may occur in patient tumors to determine markers of cell physiology and to screen anticancer therapies.

## Methods

### Cell culture

The human PANC-1 pancreatic adenocarcinoma cell line was purchased from the American Type Culture Collection (Cat# CRL-1469) and grown in high glucose Dulbecco’s Modified Eagle’s Medium with 10% fetal bovine serum (Gibco) without antibiotics. For all drug dosing experiments, harvested cells were seeded onto our custom plates that were pre-warmed to 37 °C to reduce thermal gradients in order to minimize the ‘edge effect’ often seen in 96-well plates^25^. Cells were allowed to attach for 24 h under 95% air/5% CO_2_ atmospheres before placement in the gas manifold for variable oxygen experiments.

Growth of PANC-1 cells under different oxygen conditions was determined by cell counting using automated image analysis of cells stained with 4′,6-diamidino-2-phenylindole (DAPI). The images were acquired from four different wells under identical conditions and nuclei were delineated and enumerated using NIH ImageJ software^36^ and the average and standard deviation were computed for each *p*O_2_ level. In parallel, cell proliferation was also monitored by 3-(4,5-dimethylthiazol-2-yl)-2,5- diphenyltetrazolium bromide (MTT) assay^37^. For analyzing MTT dose–response data, the MTT absorbance at each *p*O_2_ level was normalized to the absorbance reading at the zero dose for that *p*O_2_ level. To validate counting or MTT assays, cells were harvested by trypsinization, counted with a hemacytometer and seeded at levels of 5,000, 10,000 and 20,000 cells per well and allowed to attach for 6 h under normal incubator conditions. Cells were then exposed to eight different static or cycling conditions for 24 h before counting or MTT assays.

### Device fabrication

The manifolds (M1), and (M2) (Fig. 2) and lid (L) (Fig. 2) were fabricated from cast polymethylmethacrylate (acrylic) polymer sheets (McMaster-Carr, Catalog #8589K62) using a laser cutter to etch the gas channels. One mm circular holes were cut into the lid using a laser cutter for pipet access. A 2 mm thick PDMS membrane (McMaster-Carr, Catalog # 86915K16) was used to produce the cover (SC) (Fig. 2). This cover was pierced with a razor knife to facilitate pipet access to the wells. Buna-N rubber (McMaster-Carr, Catalog #8635K812) was used to fabricate the upper (UG) and lower gaskets (LG) (Fig. 2) and was cut with a laser cutter. Tygon gas tubing (VWR, catalog #89404-068, OD = 5/32 in, ID = 3/32 in) was used to recirculate gas flow from the lower to upper channels.

The permeable-bottom 96-well plates were fabricated by attaching a 125 μm thick PDMS membrane to bottomless 96-well plates (Greiner Bio-One, Catalog# 655000-06) by the procedure outlined in our earlier publications^24,25^. The PDMS membrane was coated with a 50 μg·mL^−1^ fibronectin solution (Sigma-Aldrich, Product Number F1141) for 6 h and rinsed with phosphate-buffered saline prior to use in cell studies.

### Drug treatment

Tirapazamine (Sigma-Aldrich, Catalog #27314-97-2), and doxorubicin (Sigma-Aldrich, Catalog #25316-40-9) were used to treat PANC-1 cells. 5000 PANC-1 cells were seeded in each well, allowed to attach under normoxia for 24 h and cultured in the device under the desired static or cycling low *p*O_2_ conditions for an additional 24 h. Narrow pipet tips (VWR, Catalog #37001-152) were used to access wells to remove media and to add fresh media/drug. After drug addition, the cells were cultured with drugs for 48 h. Cell proliferation was determined by MTT assay.

Two gas manifolds were used in the drug dosing studies. The first was reported in our earlier study^25^ and incorporates a mixing tree and combines two input gases to produce eight different static or cycling oxygen mixtures. This design enables four drug doses to be done in triplicate at each of the eight *p*O_2_ levels. The second, gas partitioning manifold, was reported in our later study^24^ and allows four rows of the plate to be set to one static or cycling oxygen level and the other four rows set to a second different *p*O_2_ level. This plate enables a maximum of 16 different drug doses to be done in triplicate at two *p*O_2_ levels.

### Oxygen sensing

Oxygen readings in gas mixtures, water and cell growth media were obtained using 140 μm diameter fiber optic sensors (Profiling Oxygen Microsensor, PreSens, Germany). PreSens specifications indicate a sensitivity down to *p*O_2_ = 0.4 mmHg^38^. Sensors were calibrated each day by a two-point calibration method as detailed previously^25^. Due to their fragility, the optical fiber is positioned within its needle bevel housing 100 μm short of the needle tip. Therefore, by placing the steel needle tip on the membrane at the bottom of the well, the sensor provides readings at a point 100 μm above the membrane. This partially retracted position did not measurably affect oxygen readings^25^.

### Gas flow control

Gas supplied to culture plates was delivered by mass flow controllers (Aalborg) directed by LabView-based software (National Instruments). All experiments used two feed gas mixtures for delivery to the plate mixing tree and manifold: Gas Input 1: 95% N_2_/5% CO_2_, and Gas Input 2: 95% air/5% CO_2_. For cycling experiments, gas flow rates and switching were controlled with a LabView program.

### Theoretical modeling

The designs of the gas channels, manifold, gas recycling system and 96-well plates were evaluated by modeling the theoretical gas transfer capabilities of each design by finite element analysis using COMSOL Multiphysics software (RRID: SCR_014767). The “Transport of Diluted Species” and “Laminar Flow” toolboxes in COMSOL were used to create models of oxygen transport in the gas channels, manifold and plate. The oxygen diffusion coefficients through culture media and the polymers and other materials used in this modeling are provided in Supplementary Materials, Table S5.

## Supplementary Information

Supplementary Information.

## Data Availability

The data analyzed in this study are available from the corresponding author upon request.
