# Supplementary material for: A multiwell plate-based system for toxicity screening under multiple static or cycling oxygen environments
Source: Sci Rep. 2021 Feb 17;11:4020. doi: 10.1038/s41598-021-83579-1 (PMC7890056; doi:10.1038/s41598-021-83579-1)
Supplement: Supplementary file 1 — Supplementary Information. [file 41598_2021_83579_MOESM1_ESM.pdf]

A multiwell plate-based system for toxicity screening under multiple static or cycling oxygen environments

Ming Yao, Glenn Walker, and Michael P. Gamcsik

Supplementary Material,
Figure S1

Figure S1. Cell counting of wells initially seeded with 5,000, 10,000 or 20,000 cells after 24 h of growth at eight different static pO_2 levels. Cell counts are normalized to the level found for initial seeding of 5,000 cells under pO2 = 139 mmHg. The expected ratio of 1:2:4 validates the imaged-based cell counting method.

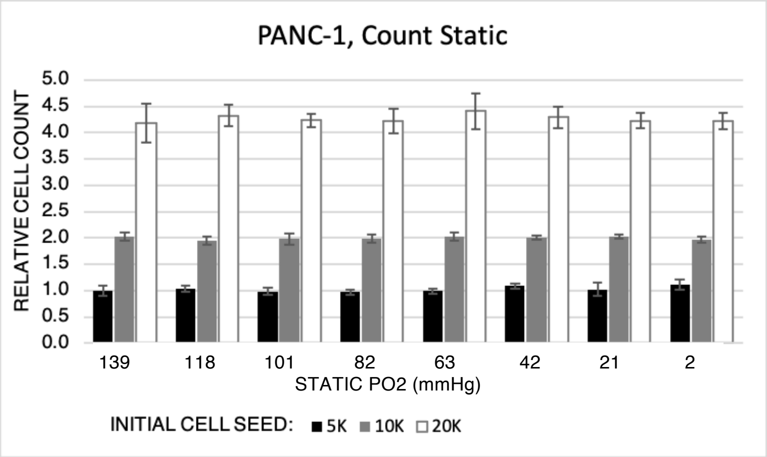

Supplementary Material, Figure S2

Figure S2. Comparison of relative MTT absorbance and cell number of PANC1 cells grown for 4 days under varying pO2 levels compared to incubator normoxia in Row A.

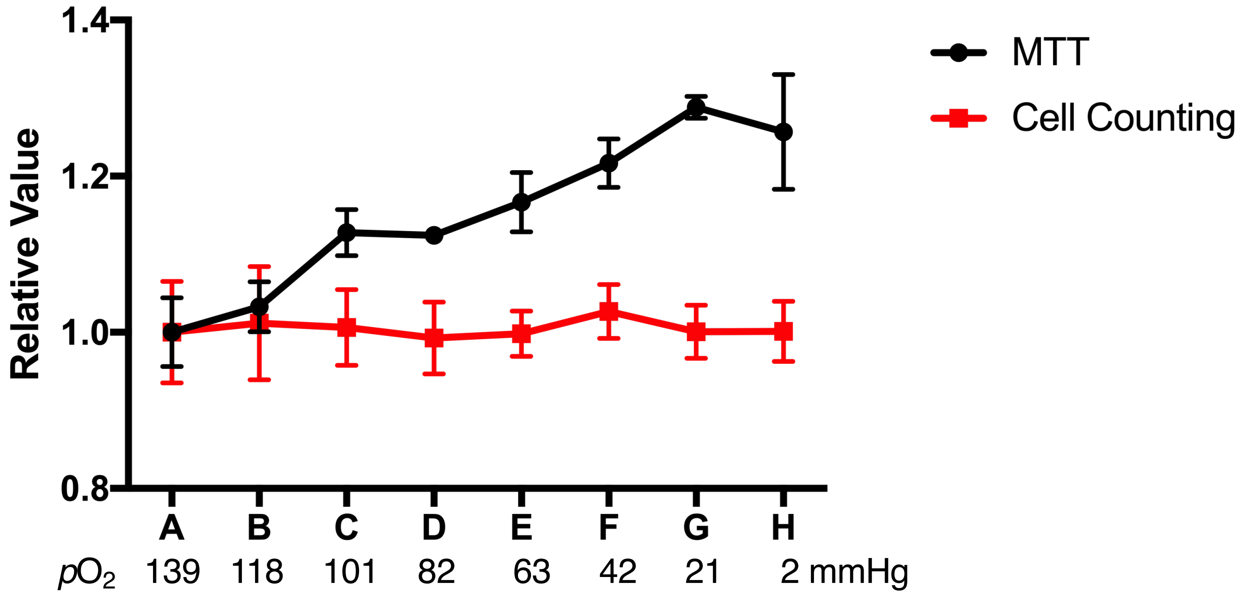

Supplementary Material, Figure S3

Figure S3. Comparison of relative MTT absorbance levels with initial cell seeding of 5,000, 10,000 and 20,000 cells after growing for 24 h under eight different static pO_2 levels. The MTT absorbance readings were normalized to the 5,000 seeding level for each pO2 environment.

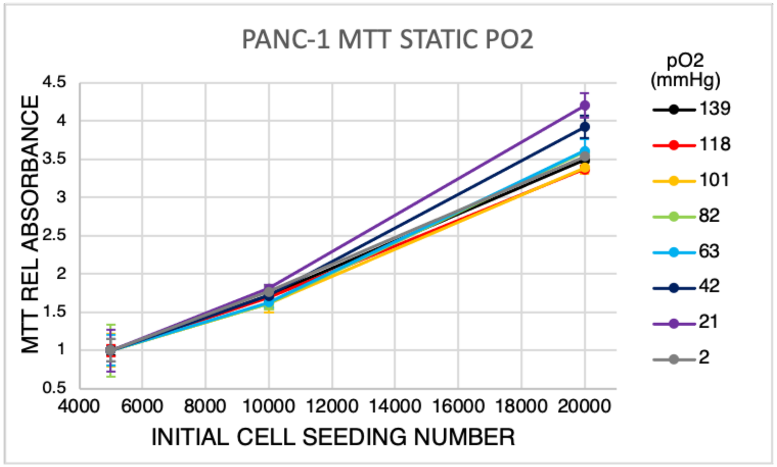

Supplementary Material, Figure S4

Figure S4. Comparison of relative MTT absorbance levels with initial cell seeding of 5,000, 10,000 and 20,000 cells after growing for 24 h under eight different cycling pO_2 patterns. The MTT absorbance readings were normalized to the 5,000 seeding level for each pO_2 environment.

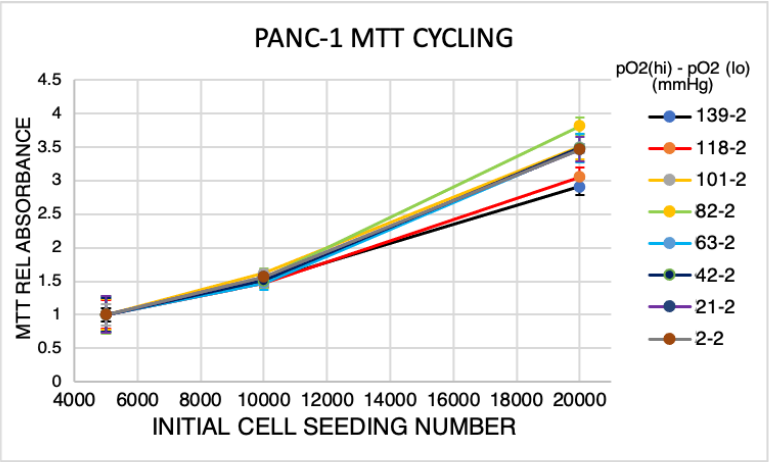

Supplementary Material, Table S5

Table S5 Parameters used in COMSOL Model

	D ($10^{-5} cm^2 \cdot sec^{-1}$)	Barrier Thickness (mm)	Component[a]
Media	2.78[1]	6.35	
PDMS	3.25[2]	0.125	Well bottom
		2.000	SC
Acrylic	0.00248[3]	4.75	M1, M2, L
Buna-N	0.006[4]	1.50	UG, LG

[a]Components as abbreviated in Fig. 2.

Data Sources:

1 Powers, D. E., Millman, J. R., Bonner-Weir, S., Rappel, M. J. & Colton, C. K. Accurate control of oxygen level in cells during culture on silicone rubber membranes with application to stem cell differentiation. *Biotechnology progress* **26**, 805-818, doi:10.1002/btpr.359 (2010).

2 Markov, D. A., Lillie, E. M., Garbett, S. P. & McCawley, L. J. Variation in diffusion of gases through PDMS due to plasma surface treatment and storage condtions. *Biomed. Microdevices* **16**, 91-96, doi:10.1007/s10544-013-9808-2 (2014).

3 Kjeldsen, P. Evaluation of gas diffusion through plastic materials used in experimental and sampling equipment. *Wat. Res.* **27**, 121-131 (1993).

4 Sturm, P. *et al.* Permeation of atmospheric gases through polymer o-rings used in flasks for air sampling. *J. Geophys. Res.* **109**, D04309, doi:10.1029/2003JD004073 (2004).
